# Wasting among Uganda men with pulmonary tuberculosis is associated with linear regain in lean tissue mass during and after treatment in contrast to women with wasting who regain fat tissue mass: prospective cohort study

**DOI:** 10.1186/1471-2334-14-24

**Published:** 2014-01-13

**Authors:** Ezekiel Mupere, LaShaunda Malone, Sarah Zalwango, Alphonse Okwera, Mary Nsereko, Daniel J Tisch, Isabel M Parraga, Catherine M Stein, Roy Mugerwa, W Henry Boom, Harriet K Mayanja, Christopher C Whalen

**Affiliations:** 1 Department of Paediatrics & Child Health College of Health Sciences, Makerere University Kampala, Kampala, Uganda; 2 Tuberculosis Research Unit, Case Western Reserve University, Cleveland, USA; 3 Uganda-Case Western Reserve Research Collaboration, Kampala, Uganda; 4 Department of Epidemiology & Biostatistics, Case Western Reserve University, Cleveland, USA; 5 Department of Nutrition, Case Western Reserve University, Cleveland, USA; 6 Department of Internal Medicine College of Health Sciences, Makerere University, Kampala, Uganda; 7 Department of Epidemiology & Biostatistics College of Public Health, University of Georgia Athens, Athens, USA

**Keywords:** Tuberculosis, HIV, Wasting, Lean tissue mass index, Fat mass index, Body mass index, Gender

## Abstract

**Background:**

Nutritional changes during and after tuberculosis treatment have not been well described. We therefore determined the effect of wasting on rate of mean change in lean tissue and fat mass as measured by bioelectrical impedance analysis (BIA), and mean change in body mass index (BMI) during and after tuberculosis treatment.

**Methods:**

In a prospective cohort study of 717 adult patients, BMI and height-normalized indices of lean tissue (LMI) and fat mass (FMI) as measured by BIA were assessed at baseline, 3, 12, and 24 months.

**Results:**

Men with wasting at baseline regained LMI at a greater rate than FMI (4.55 kg/m^2^ (95% confidence interval (CI): 1.26, 7.83 versus 3.16 (95% CI: 0.80, 5.52)) per month, respectively during initial tuberculosis therapy. In contrast, women with wasting regained FMI at greater rate than LMI (3.55 kg/m^2^ (95% CI: 0.40, 6.70) versus 2.07 (95% CI: -0.74, 4.88)), respectively. Men with wasting regained BMI at a rate of 6.45 kg/m^2^ (95% CI: 3.02, 9.87) in the first three months whereas women, had a rate of 3.30 kg/m^2^ (95% CI: -0.11, 6.72). There were minimal changes in body composition after month 3 and during months 12 to 24.

**Conclusion:**

Wasted tuberculosis patients regain weight with treatment but the type of gain differs by gender and patients may remain underweight after the initial phase of treatment.

## Background

A cardinal feature of tuberculosis patients is marked wasting at time of disease diagnosis [1-3]. Wasting associated with tuberculosis is likely caused by a combination of decreased appetite and altered metabolism resulting from inflammatory and immune responses [4-6]. Decreased appetite compounds the increased metabolic demands of the chronic inflammation associated with tuberculosis. Although antituberculosis treatment is highly effective [7], many patients remain underweight after 6 months of treatment [8] suggesting that full recovery takes longer than drug treatment itself.

In patients with both tuberculosis and HIV co-infection, wasting may be exacerbated [6,9]. Findings from some cross-sectional studies show no large differences in body composition between HIV sero-positive and sero-negative adults with tuberculosis at presentation [10-13] although gender may be a confounding factor [1,12].

Although several reports have described the nutritional changes at the time of tuberculosis diagnosis using both anthropometry and bioelectrical impedance analysis (BIA) [4,10,12,14-16], follow-up studies to explain nutritional changes during and after tuberculosis treatment are limited. Yet such information would provide the basis for designing nutritional interventions to rescue the wasting associated with tuberculosis.

Previous studies [1,17] found weight gain to be a poor indicator of clinical response in tuberculosis. Moreover, weight or body mass index (BMI) provides limited information about nutritional alterations in a patient. Measurement of body composition is necessary to obtain a better picture of the nutritional status in tuberculosis because body compartments differ in their contribution to weight gain and to clinical benefit. Lean tissue mass and fat mass as body composition measurements have been shown to permit a more precise evaluation of nutritional status [18,19]. Lean tissue mass is more closely correlated with quality of life, physical functioning and survival than are fat mass and body weight [20-22]. BIA is a useful non-invasive and cost-effective method for clinical assessment of lean tissue and fat mass despite its limitations [23,24]. In this large retrospective study, we have demonstrated the effect of baseline wasting and HIV infection on rate of change in lean tissue mass, fat mass, and BMI during and after treatment among tuberculosis patients in urban Uganda, Kampala.

## Methods

### Study design

We conducted a prospective cohort study of 745 adults (age ≥ 18 years) with initial episode pulmonary tuberculosis, defined baseline body wasting and confirmed HIV status. The data were generated from subjects enrolled in the Household Contact study [25-27] and in the placebo arm of a randomized placebo-controlled clinical trial that used prednisolone as an adjunct treatment among HIV patients with tuberculosis [28]. Data for the intervention arm of this trial was not used in the present study because prednisolone has adverse nutritional effects.

The institutional review boards at Case Western Reserve University in the United States and Joint Clinical Research Center in Uganda reviewed the protocol and final approval was obtained from the Uganda National Council for Science and Technology. All participants provided written informed consent for enrollment into the study and were given appropriate pre- and post-test HIV counseling and AIDS education.

At enrollment, basic demographic information and medical history were collected by self report, and a standardized physical examination was conducted by a medical officer. Active tuberculosis was confirmed by sputum smear microscopy and culture. Patients with active tuberculosis were treated with standard four-drug chemotherapy for tuberculosis in accordance with the Ugandan Ministry of Health guidelines. HIV-1 infection was diagnosed on the basis of a positive enzyme-linked immunosorbent assay for HIV-1 antibodies (Recombigen; Cambridge Biotech, Cambridge, MA). BMI was available at baseline and follow-up for all subjects whereas BIA baseline and follow-up data were available for only a subset of 278 participants from the household contact study.

Nutritional status was assessed using anthropometric measurements (height and weight) and BIA (RJL Systems, Quantum II, Detroit, MI). Weight was determined to the nearest 0.1 kg using a SECA adult balance scale, and standing height was determined to the nearest centimeter. Anthropometric and BIA measurements were performed on the day patients were confirmed to have tuberculosis disease and at scheduled visits including baseline, 3, 12, and 24 months on follow-up using the same equipment and standard conditions [23]. Body-mass index (BMI) was computed using the relationship of weight in kilograms divided by height in meters squared (kg/m^2^).

Single-frequency BIA was performed at 50 kHz and 800 mA with standard tetrapolar lead placement [29]. Before performing measurements, the BIA instrument was calibrated using the manufacturer’s recalibration device. The resistance and reactance were based on measures of a series circuit [30]. BIA measurements were performed in triplicate for each subject after resting the participant for a minimum of 2 hours, having voided their bladder, no alcohol intake in previous 24 hours and at ambient temperature. Average measurements were used in the analysis. Lean tissue mass was calculated from BIA measurements using equations that were previously cross-validated in a sample of patients (white, black and Hispanic) with and without HIV infection [30] and have been applied elsewhere in African studies [10,15,16]. Fat mass was calculated as body weight minus lean tissue mass.

We used BMI and height-normalized indices (adjusted for height^2^) of body composition that partition BMI into lean tissue mass index (LMI) and fat mass index (FMI) [18,19,31] to establish the body wasting status of participants. The LMI and FMI have the advantages of compensating for differences in height and age [24]. Also, use of LMI and FMI eliminates some of the differences between population groups. We defined body wasting as patients having low LMI (<16.7 (kg/m^2^) for men and <14.6 (kg/m^2^) for women) and low FMI (<1.8 (kg/m^2^) for men and <3.9 (kg/m^2^) for women) [19] corresponding to WHO BMI categories (<18.5 kg/m^2^) for malnutrition among adults as previously reported [32].

### Statistical analysis

The main aim of the analysis was to examine the change in body composition during and after tuberculosis treatment as measured by LMI, FMI, and BMI; and to relate these changes to baseline wasting. Based on our previous findings [12,22], we hypothesized that rate of change in LMI, FMI, and BMI would differ in patients with or without baseline wasting and it would also differ by sex.

The main outcome vectors of the participant’s LMI, FMI, and BMI were modeled as linear combinations of baseline covariates and baseline wasting status in separate multilevel linear random mixed models with random intercept and random slope, accounting for correlation of repeated measurements within each individual [33-35]. We used the full maximum likelihood estimation method for parameter estimation and type 3 F-test for testing significance. We assumed a spatial exponential covariance structure because the intervals between serial data points were different by design. Piecewise linear mixed models [36,37] were used to assess, for a given measure, the change in gradient over time at the knot for month 3 and at month 12. The knots in the piecewise were chosen a priori basing on the scheduled data point measurements. The main predictor was baseline wasting and the following were other covariates used to assess associations with the dependent variables overtime: HIV sero-status; anemia (hemoglobin ≤10 mg/dl); prior smoking status; history of weight loss; and extent of disease on chest x-ray categorized as normal/minimal and moderate/far advanced. The interval between study visits was adjusted to an annual scale to facilitate the interpretation of rates of LMI, FMI, and BMI decline per year.

Data missingness was assumed not to be informative on outcome variable and ignorable after performing separate generalizing estimating equations models for LM, FMI, and BMI using Proc GENMOD with a logit link function to assess the effect of having wasting versus no wasting at baseline on to probability of missing overtime [38]. To assess for the confounding effects of HIV and gender, we fitted stratified models according to HIV strata and gender. The analyses were performed using SAS MIXED procedures [39] and SAS version 9.1.3 Cary software, North Carolina SAS Institute Inc. 2004.

## Results

### Baseline characteristics of the study population

Of the 745 participants who were available, 28 were excluded due to lack of anthropometric follow-up data, leaving 717 participants in total for analysis. Of the 717 participants, 628 were from the household contact study and 89 from the placebo arm of the randomized controlled trail. The household contact and the placebo arm of the randomized controlled trial participants had comparable baseline characteristics including gender (p = 0.109), wasting as measured by BMI (p = 0.286), and proportion of anemic individuals (hemoglobin ≤10 mg/dl) (p = 0.170); however, differences were observed for current smoking status (p = 0.020), and extent of disease on chest radiography (p = 0.004). All the 717 participants had serial BMI measurements and 13% (92/717) of the 717 died on follow-up. A subset 278 from the household contact study of the 717 participants had LMI and FMI follow-up data for analysis. Of the 278 who had BIA data, 8% (22/278) died on follow-up. The 717 participants and the 278 subset of participants with BIA data had comparable baseline characteristics including wasting as measured by BMI (p = 0.45), gender (p = 0.56), proportion of anemic individuals (hemoglobin ≤10 mg/dl) (p = 0.59), current smoking status (p = 0.95), and extent of disease on chest radiography (p = 0.13).

Of the 717 patients who were included in the analysis, 293 (41%) were wasted as defined by BMI <18.5 kg/m^2^ at baseline (Table 1). Of the 278 participants with BIA baseline and follow-up data, 94 (34%) were wasted as measured by LMI. Of the 278 patients, 120 (43%) were wasted as measured by FMI (Table 1). There were gender differences in wasting status. Men had significantly greater reductions in LMI and BMI compared to women whereas women had significantly greater reductions in FMI compared to men (Table 1). There were no differences in wasting between HIV positive and HIV negative patients by any of the nutritional assessment measurements.

**Table 1:** Baseline characteristics of tuberculosis patients with/or without baseline wasting in urban Uganda, Kampala

**Characteristic**	**LMI**		**FMI**		**BMI**	
	**No wasting**	**Wasting**	**No wasting**	**Wasting**	**No wasting**	**Wasting**
	**(n = 184)**	**(n = 94)**	**(n = 158)**	**(n = 120)**	**(n = 424)**	**(n = 293)**
Age (years)						
≤30 (%)	122 (66)	54 (57)	99 (63)	77 (64)	252 (59)	170 (58)
>30 (%)	62 (34)	40 (43)	59 (37)	43 (36)	172 (41)	123 (42)
Sex						
Female (%)	115 (62)	19 (20)^a^	65 (41)	69 (58)^b^	232 (55)	115 (39)^a^
Male (%)	69 (38)	75 (80)	93 (59)	51 (42)	192 (45)	178 (61)
HIV-serostatus						
Negative (%)	105 (57)	53 (56)	90 (57)	68 (57)	198 (47)	140 (48)
Positive (%)	79 (43)	41 (44)	68 (43)	52 (43)	226 (53)	153 (52)
Hemoglobin (mg/dl)^1^						
>10 (%)	140 (76)	72 (77)	135 (85)	77 (64)^a^	208 (81)	118 (64)^a^
≤10 (%)	44 (24)	22 (23)	23 (15)	43 (36)	48 (19)	67 (36)
Smoker^2^						
No (%)	159 (87)	56 (60)^a^	127 (80)	88 (74)	364 (86)	210 (72)^a^
Yes (%)	24 (13)	38 (40)	31 (20)	31 (26)	58 (14)	82 (28)
Takes alcohol^3^						
No (%)	135 (74)	59 (63)	106 (67)	88 (74)	259 (61)	192 (66)
Yes (%)	48 (26)	35 (37)	52 (33)	31 (26)	164 (39)	101 (34)
Extent on chest x-ray^4^						
Normal/minimal (%)	38 (21)	8 (9)^b^	26 (17)	20 (17)	74 (18)	33 (11)^b^
Moderate/far advanced (%)	143 (79)	86 (91)	131 (83)	98 (83)	346 (82)	257 (89)
Weight loss^5^						
No (%)	32 (17)	12 (13)	31 (20)	13 (11)	107 (25)	52 (18)^b^
Yes (%)	151 (83)	82 (87)	126 (80)	107 (89)	315 (75)	240 (82)

^a^p-value <0.001, ^b^p-value <0.05; p-values obtained by chi-square test. BMI = body mass index, LMI = Lean tissue mass index, FMI = fat mass index. ^1^276 missed hemoglobin measurement due to lack of blood for BMI data; ^4^three missed extent variable in BIA data and 7 in BMI data; ^3^one missed history of prior smoking in BIA data and 3 missed in BMI data; ^3^one missed history of alcohol intake for BMI and BIA data; ^5^one missed history of weight loss for BIA data and 3 missed in BMI data; LMI wasting (<16.7 kg/m^2^ for men and <14.6 kg/m^2^ for women, normal (≥ 16.7 kg/m^2^ for men, ≥14.6 kg/m^2^ for women); reduced BMI <18.5 kg/m^2^.

### Impact of wasting and gender on rate of mean change in LMI during and after treatment

**Figure 1:**
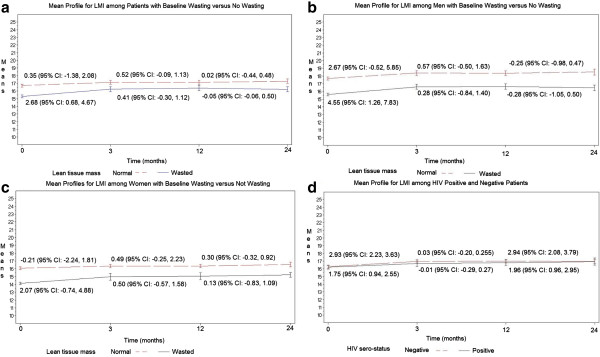
Panels show mean profiles for LMI among patients with baseline lean tissue wasting versus no wasting (a); among men with baseline wasting versus no wasting (b); among women with baseline wasting versus no wasting (c); and among HIV positive versus negative patients (d).

The effect of wasting as measured by LMI was associated with a significant gain in LMI at a rate of 2.68 (95% CI: 0.68, 4.67) kg/m^2^ per month during the first three months of treatment after adjusting for HIV, status of anemia, prior smoking status, history of weight loss, and extent of disease on chest x-ray (Figure 1a; Additional file 1: Table S1 and Additional file 2: Table S2). Patients who presented with no wasting had gradual increase in LMI at a rate of 0.36 (95% CI: -1.38, 2.08) kg/m^2^ per month. There were gradual changes in LMI after three months and after treatment regardless of wasting status at baseline (Figure 1a; Additional file 1: Table S1 and Additional file 2: Table S2). Patients who presented with wasting did not regain LMI to the same level as those who presented without wasting.

When we stratified to test for the confounding effects of gender and HIV on gain in LMI over time, men had a higher LMI at presentation than women. Men with wasting regained LMI at a greater rate of 4.55 (95% CI: 1.26, 7.83) kg/m^2^ than women at 2.07 (95% CI: -0.74, 4.88) kg/m^2^ per month during the first 3 months of treatment (Figure 1b and c; Additional file 1: Table S1 and Additional file 2: Table S2). A similar pattern was seen among patients without wasting at baseline (Figure 1b and c; Additional file 1: Table S1 and Additional file 2: Table S2). After the first 3 months of treatment, LMI gain was reduced in both men and women. Both HIV sero-positive and sero-negative patients regained LMI over first 3 months and after three months; however, there was comparable tendency for HIV sero-negative patients to regain at the same rate as HIV sero-positive patients (Figure 1d).

### Impact of wasting and gender on rate of mean change in FMI during and after treatment

**Figure 2:**
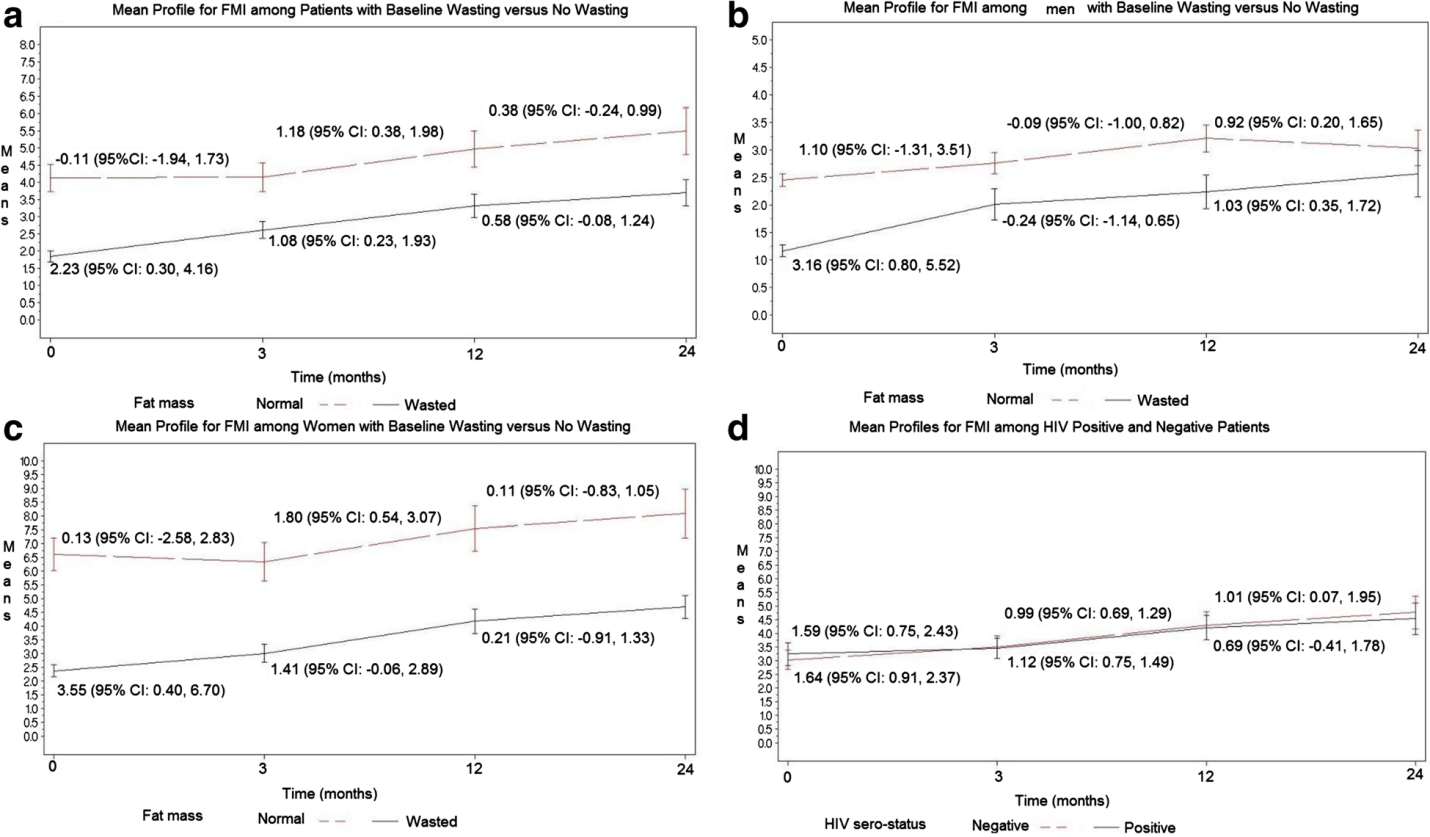
Panels show mean profiles for FMI among patients with baseline fat wasting versus no wasting (a); among men with baseline wasting versus no wasting (b); among women with baseline wasting versus no wasting (c); and among HIV positive versus negative patients (d).

Patients regained FMI over time, up to 24 months regardless of baseline fat wasting as measured by low FMI; however, there was a tendency for patients with baseline fat wasting to gain FMI at higher rate compared to those with preserved fat tissue (Figure 2a; Additional file 1: Table S1 and Additional file 2: Table S2). During the first three months, patients who presented with wasting had a significant gain in FMI at rate of 2.23 (95% CI: 0.30, 4.16) kg/m^2^ per month whereas patients who presented with normal fat mass had minimal change at a rate of −0.11 (95% CI: -1.98, 1.73) kg/m^2^ per month after adjusting for HIV, prior smoking status, history of weight loss, and extent of disease on chest x-ray (Figure 2a; Additional file 1: Table S1 and Additional file 2: Table S2). There were minimal changes in FMI after month 3 and before month 12 among women and men regardless of initial fat mass wasting status.

During the first three months in stratified analysis (Figure 2b and c; Additional file 1: Table S1 and Additional file 2: Table S2), women had higher FMI at diagnosis than men. Among patients who presented with wasting, women gained more FMI at rate of 3.55 (95% CI: 0.40, 6.70) kg/m^2^ per month compared to men at rate of 3.16 (95% CI: 0.80, 5.52). Regarding HIV status, there were minimal changes in FMI regardless of the initial fat mass level suggesting that both HIV sero-positive and sero-negative patients regain FMI at similar rates (Figure 2d).

### Impact of wasting and gender on rate of mean change in BMI during and after treatment

**Figure 3:**
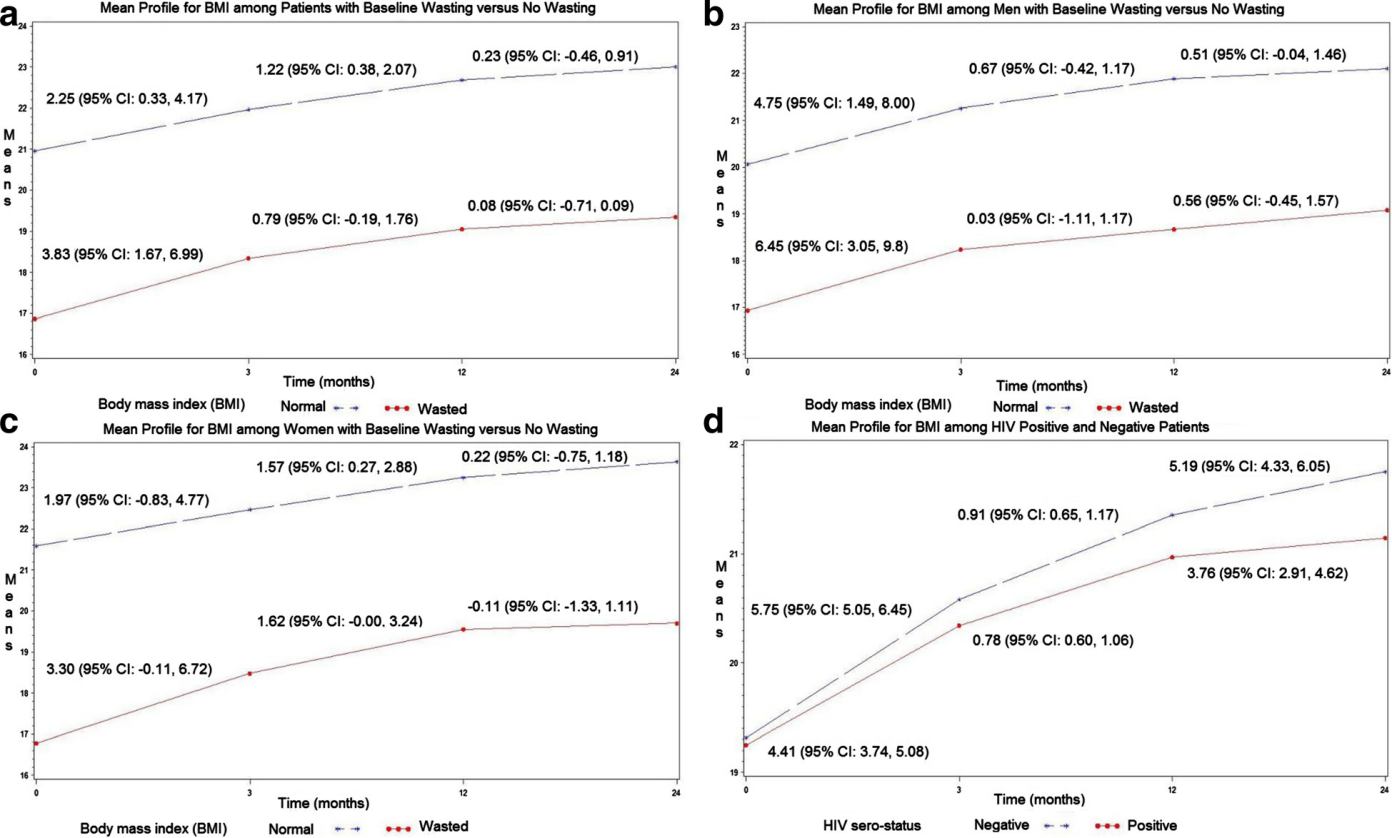
Panels show mean profiles for BMI among patients with baseline body mass wasting versus no wasting (a); among men with baseline wasting versus no wasting (b); among women with baseline wasting versus no wasting (c); and among HIV positive versus negative patients (d).

During the first three months, BMI gain was at the rate of 3.83 (95% CI: 1.67, 5.99) kg/m^2^ per month among patients who presented with wasting compared to the rate of 2.25 (95% CI: 0.33, 4.17) kg/m^2^ per month among patients who presented with no wasting after adjusting for HIV, status of anemia, prior smoking status, history of weight loss, and extent of disease on chest x-ray (Figure 3a; Additional file 1: Table S1). The magnitude in BMI gain among patients who presented with wasting was higher in men at rate of 6.45 (95% CI: 3.02, 9.87) and minimal in women at a rate of 3.30 (95% CI: -0.11, 6.72) (Figure 3b and c; Additional file 1: Table S1 and Additional file 2: Table S2). Similar results were obtained among the 89 participants from the placebo arm of the randomized controlled trail. There were gradual changes in BMI after month 3 and during the one year of follow-up after month 12 in the overall population and in stratified models for women and men (Figure 3b and c; Additional file 1: Table S1 and Additional file 2: Table S2). There were minimal differences in BMI gain between HIV sero-positive and sero-negative patients over the entire 24 months period (Figure 3d).

## Discussion

In this prospective cohort study of 717 adults who had BMI measurements and in the subset of 278 of the 717 who had BIA measurements in urban Uganda, body wasting was associated with a dramatic linear gain in BMI and gain in both LMI and FMI in the initial three months of tuberculosis treatment, respectively. The rate of increase varied by gender but not by HIV status of the patient. Men with wasting at baseline regained lean tissue mass at a greater rate than fat tissue mass during initial tuberculosis therapy. In contrast, women with wasting regained fat mass at greater rate than lean tissue mass. Men without wasting regained lean tissue mass without substantial gain in fat mass, whereas women without wasting had minimal changes in lean tissue or fat mass. There were minimal changes in body composition after three months of tuberculosis treatment and during the one year period of follow-up after month 12 among regardless of the initial body composition, gender and HIV status. This suggests that patients remain underweight after 3 months of treatment. Full nutritional recovery takes much longer than treatment itself, and may never fully catch-up in those who at the time of tuberculosis diagnosis were wasted as previously reported [40]. It is also possible that a substantial proportion of patients with potential to regain in body composition might have died because of marked wasting at time of tuberculosis diagnosis [20-22]. HIV appears to play a marginal role for changes in body composition in co-infected individuals.

This study is the first to report that the initial LMI and BMI influence subsequent changes in body composition among men with tuberculosis. Results of the present study are consistent with previous studies [41] that revealed total lean tissue increased during the first six weeks of tuberculosis treatment and thereafter the increase was in the fat mass compartment. In our present study, there were minimal changes in body composition after month 3 including slight increase, in fat mass among men. The prior study [41]; however, was limited in sample size with only 36 participants because it was a prospective randomized clinical trial. The study population comprised of only patients who were wasted (BMI <18.5 kg/m^2^), had no HIV comparison group, and followed patients for only 6 months. The strengths of the present study include: large number of study participants comprising both women and men, wasted and not wasted persons, HIV positive and HIV negative individuals, and long duration of follow-up.

The present study demonstrates that patients who presented with reduced fat and lean tissue mass gained in body composition at a higher rate than patients who presented with normal levels of fat and lean tissue mass, particularly during the first three months of tuberculosis treatment. However, the gain was predominantly fat mass among women whereas men gained predominantly lean tissue mass plus substantial levels of fat mass. The potential explanation appears to rest on the gender differences in lean tissue mass content. Women tend to have low lean tissue mass compared to men, yet lean tissue mass has been shown to be a significant determinant of fat oxidation [42,43]. In several reports after adjusting for differences in lean tissue mass, resting fat oxidation is lower in women than men [42,44,45]. Thus, this gender differences in fat metabolism as explained elsewhere [46-48], is associated with lower basal fat oxidation that may contribute to the increased fat storage or gain in women compared to men. Moreover, longitudinal studies have shown that low rates of fat utilization predict subsequent weight [49]. Besides differences in lipolysis, other factors such as differences in hormone action may contribute to differences in fat oxidation. Higher levels of androgens in men stimulate the formation of the nucleic acids essential for protein biosynthesis [50] with eventual increase in lean tissue and recent studies [51] postulate that major sex hormones such estrogen and testosterone are central to weight management.

In normal healthy adults, protein catabolism equals protein anabolism; however, protein catabolism exceeds protein anabolism in tuberculosis [52]. In general; however, the linear increase in lean tissue during the first three months among patients who presented with reduced lean tissue mass levels suggest that patients with tuberculosis can mount a protein anabolic response during treatment once bacterial burden is reduced and controlled [5,52]. It may be possible that as the patient becomes sterile from the causative organism during treatment, the degree of body wasting offsets the tuberculosis effect on protein catabolism until a net state of anabolism [53]. Alternatively, the response could be explained by the effective adaptive response of protein metabolism to a chronic inflammatory state [54].

Despite the dramatic linear increase in lean tissue mass, fat mass, and BMI among patients who presented with body wasting, these patients did not normalize their indices for body composition. Wasted patients did not regain body composition comparable to patients presenting without wasting. There may be several explanations for this. It is possible that patients who present with wasting at the time of tuberculosis diagnosis were of slim body build before disease as compared to the patients who present with normal nutritional status as previously reported that patients with slim body build were at increased risk for tuberculosis [55]. While on tuberculosis treatment, the patients with body wasting apparently regain the original body composition status prior to onset of tuberculosis disease. Following attainment of the original body composition, there are minimal changes in lean tissue and individuals adapt effective energy-sparing mechanism in balance with the usual energy intake [56,57]. It is also possible that households where tuberculosis occurs in Uganda, and in other countries in sub-Saharan region, there is food insecurity compromising energy intake. Thus, persistently low body composition may mark lack of access to sufficient calorie, protein, and other nutrient intake. It is also possible that tuberculosis causes permanent loss of lean tissue that cannot be regained. The persistently low body composition measures even after effective tuberculosis treatment may be a marker for future health risk. Among patients with wasting who do not normalize, there may be consequences regarding survival and physical function in response to future disease insults. Further research is needed to understand the health risk among these patients.

In this study, we used the BIA method to measure body composition, yet it is not a reference standard like dual-energy x-ray absorptiometry. The BIA prediction method used has not yet been validated in the local population. As a result, findings of body composition may be biased because of variations in hydration across ethnic groups [23]. However, the equations that were used in this study were previously cross validated in individuals of different races (white, black, and Hispanic) among men and women, who were both healthy controls and HIV-infected patients [30]. Moreover, the equations have been used widely in other studies from Africa with meaningful findings [10,12,15,16]. Further, our findings are similar to studies that used state-of-the art reference methods [58]. We also took care to take measurements at rest, with proper placement of leads, in participants who had not exercised or taken alcohol, in participants with voided bladder and ambient temperature. However, measurements were in patients with underlying illness that may cause shifts in body water compartments, thereby affecting measurements of fat mass. Our findings are also limited by the lack of dietary intake assessment to give further insight in the interpretation of gender differences in longitudinal body composition changes and we did not evaluate HIV disease severity.

## Conclusions

Despite limitations of the present study, this study suggests that the body regains weight among wasted tuberculosis patients. Patients with wasting do not recover to the same levels of body mass as those who were not wasted. This suggests that tuberculosis leads to permanent loss of lean tissue and fat mass. There was remarkable gender but not HIV differences in longitudinal body composition changes during the initial phase of tuberculosis treatment among patients who presented with body wasting. The substantial body composition differences between men and women suggest that the nutritional demands during recovery may differ for men and women. Evaluation of nutritional status should involve evaluation of lean tissue and fat mass and further studies should explore the impact of providing nutritional interventions as adjuvant treatment on body composition among tuberculosis patients during and after treatment in sub-Saharan Africa.

## Abbreviations

HIV: Human immunodeficiency virus; BMI: Body mass index; LMI: Lean tissue mass index; FMI: Fat mass index; BIA: Bioelectrical impedance analysis; CI: Confidence interval; AIDS: Acquired Immunodeficiency syndrome.

## Competing interest

The authors declare that they have no competing interests.

## Authors’ contribution

Authors made the following contributions: EM, DJT, IMP, RM, WHB, HMK, and CCW designed research; EM, LM, SZ, AO, MN, CMS, RM, WHB, HMK, and CCW conducted research and analyzed data; EM, LM, SZ, AO, MN, DJT, IMP, CMS, RM, WHB, HMK, and CCW wrote the paper; and EM, LM, CMS, WHB, and CCW had primary responsibility for final content. All authors read and approved the final manuscript.

## Pre-publication history

The pre-publication history for this paper can be accessed here:

http://www.biomedcentral.com/1471-2334/14/24/prepub

## Supplementary Material

Additional file 1: Table S1 Impact of lean tissue, fat and body mass wasting on rate of mean change in LMI, FMI and BMI during and after pulmonary tuberculosis treatment in Kampala, Uganda. Click here for file

Additional file 2: Table S2 Changes in lean tissue mass, fat mass and body mass index during and after tuberculosis treatment among adult patients with or without baseline wasting in Kampala, Uganda. Click here for file
