# Supplementary material for: Wasting among Uganda men with pulmonary tuberculosis is associated with linear regain in lean tissue mass during and after treatment in contrast to women with wasting who regain fat tissue mass: prospective cohort study
Source: BMC Infect Dis. 2014 Jan 13;14:24. doi: 10.1186/1471-2334-14-24 (PMC3922730; doi:10.1186/1471-2334-14-24)
Supplement: Additional file 1: Table S1 — Impact of lean tissue, fat and body mass wasting on rate of mean change in LMI, FMI and BMI during and after pulmonary tuberculosis treatment in Kampala, Uganda. [file 1471-2334-14-24-S1.doc]

**Additional file 1**

**Table S1: Impact of lean tissue, fat and body mass wasting on rate of mean change in LMI, FMI and BMI during and after pulmonary tuberculosis treatment in Kampala,** Uganda

| **Characteristics** |  |  | |  | | **Stratified models** | | | | | |
| --- | --- | --- | --- | --- | --- | --- | --- | --- | --- | --- | --- |
|  | **Overall model** | | | | | **Women** | | | **Men** | | |
|  | **Rate** | | **SE** | | **95% CI** | **Rate** | **SE** | **95% CI** | **Rate** | **SE** | **95% CI** |
| **Baseline lean tissue mass** |  | |  | |  |  |  |  |  |  |  |
| Not wasted, slope before 3 mo | 0.35 | | 0.88 | | -1.38, 2.08 | -0.21 | 1.03 | -2.24, 1.81 | 2.67 | 1.62 | -0.52, 5.85 |
| Wasted, slope before 3 mo | 2.68 | | 1.05 | | **0.68, 4.67** | 2.07 | 1.43 | -0.74, 4.88 | 4.55 | 1.67 | **1.26, 7.83** |
| Not wasted, slope after 3 mo | 0.52 | | 0.31 | | -0.09, 1.13 | 0.49 | 0.38 | -0.25, 2.23 | 0.57 | 0.54 | -0.50, 1.63 |
| Wasted, slope after 3 mo | 0.41 | | 0.36 | | -0.30, 1.12 | 0.50 | 0.55 | -0.57, 1.58 | 0.28 | 0.57 | -0.84, 1.40 |
| Not wasted, slope after 12 mo | 0.02 | | 0.23 | | -0.44, 0.48 | 0.30 | 0.31 | -0.32, 0.92 | -0.25 | 0.37 | -0.98, 0.47 |
| Wasted, slope after 12 mo | -0.05 | | 0.28 | | -0.60, 0.50 | 0.13 | 0.49 | -0.83, 1.09 | -0.28 | 0.39 | -1.05, 0.50 |
| **Baseline fat mass** |  | |  | |  |  |  |  |  |  |  |
| Not wasted, slope before 3 mo | -0.11 | | 0.93 | | -1.94, 1.73 | 0.13 | 1.38 | -2.58, 2.83 | 1.10 | 1.23 | -1.31, 3.51 |
| Wasted, slope before 3 mo | 2.23 | | 0.98 | | **0.30, 4.16** | 3.55 | 1.60 | **0.40, 6.70** | 3.16 | 1.20 | **0.80, 5.52** |
| Not wasted, slope after 3 mo | 1.18 | | 0.41 | | **0.38, 1.98** | 1.80 | 0.64 | **0.54, 3.07** | -0.09 | 0.46 | -1.00, 0.82 |
| Wasted, slope after 3 mo | 1.08 | | 0.43 | | **0.23, 1.93** | 1.41 | 0.75 | -0.06, 2.89 | -0.24 | 0.46 | -1.14, 0.65 |
| Not wasted, slope after 12 mo | 0.38 | | 0.31 | | -0.24, 0.99 | 0.11 | 0.48 | -0.83, 1.05 | 0.92 | 0.37 | **0.20, 1.65** |
| Wasted, slope after 12 mo | 0.58 | | 0.34 | | -0.08, 1.24 | 0.21 | 0.57 | -0.91, 1.33 | 1.03 | 0.35 | **0.35, 1.72** |
| **Baseline body mass** |  | |  | |  |  |  |  |  |  |  |
| Not wasted, slope before 3 mo | 2.25 | | 0.98 | | **0.33, 4.17** | 1.97 | 1.43 | -0.83, 4.77 | 4.75 | 1.66 | **1.49, 8.00** |
| Wasted, slope before 3 mo | 3.83 | | 1.10 | | **1.67, 5.99** | 3.30 | 1.74 | -0.11, 6.72 | 6.45 | 1.75 | **3.02, 9.87** |
| Not wasted, slope after 3 mo | 1.22 | | 0.44 | | **0.36, 2.07** | 1.57 | 0.67 | **0.27, 2.88** | 0.67 | 0.56 | -0.42, 1.76 |
| Wasted, slope after 3 mo | 0.79 | | 0.49 | | -0.17, 1.76 | 1.62 | 0.83 | -0.00, 3.24 | 0.03 | 0.58 | -1.11, 1.17 |
| Not wasted, slope after 12 mo | 0.23 | | 0.35 | | -0.46, 0.91 | 0.22 | 0.49 | -0.75, 1.18 | 0.51 | 0.48 | -0.43, 1.46 |
| Wasted, slope after 12 mo | 0.08 | | 0.40 | | -0.71, 0.87 | -0.11 | 0.62 | -1.33, 1.11 | 0.56 | 0.51 | -0.45, 1.57 |

LMI = Lean tissue mass index; FMI = Fat mass index; and BMI = Body mass index. LMI and BMI multivariable models were adjusted for HIV, status of anemia, prior smoking status, history of weight loss, and extent of disease on chest x-ray. FMI multivariable model was adjusted for HIV, prior smoking status, history of weight loss, and extent of disease on chest x-ray. Lean tissue mass wasting = LMI <16.7 kg/m^2^ for men and <14.6 kg/m^2^ for women, fat mass wasting = FMI <1.8 kg/m^2^ for men and <3.9 kg/m^2^ for women; reduced BMI <18.5 kg/m^2^ for women and men.
