# Supplementary material for: Wasting among Uganda men with pulmonary tuberculosis is associated with linear regain in lean tissue mass during and after treatment in contrast to women with wasting who regain fat tissue mass: prospective cohort study
Source: BMC Infect Dis. 2014 Jan 13;14:24. doi: 10.1186/1471-2334-14-24 (PMC3922730; doi:10.1186/1471-2334-14-24)
Supplement: Additional file 2: Table S2 — Changes in lean tissue mass, fat mass and body mass index during and after tuberculosis treatment among adult patients with or without baseline wasting in Kampala, Uganda. [file 1471-2334-14-24-S2.doc]

**Additional file 2: Table S2: Changes in Lean Tissue Mass, Fat Mass and Body Mass Index During and After Tuberculosis Treatment Among Adult Patients with or without Baseline Wasting in Kampala, Uganda**

| **Characteristic** | **Men** | | | | **Women** | | | |
| --- | --- | --- | --- | --- | --- | --- | --- | --- |
|  | Month 0  (n=166) | Month 3  (n=126) | Month 12  (n=115) | Month 24  (n=91) | Month 0  (n=147) | Month 3  (n=115) | Month 12  (n=100) | Month 24  (n=92) |
| **Lean tissue mass (LM)** |  |  |  |  |  |  |  |  |
| LM with no baseline wasting | 49.8 ± 4.5 | 52.0 ± 5.5 | 51.7 ± 4.4 | 52.5 ±5.8 | 39.9 ± 4.2 | 40.8 ± 4.2 | 40.8 ± 4.1 | 41 ± 4.4 |
| ^1^Percent change in LM with no baseline wasting | - | 3.8 ± 6.6 | 0.7 ± 5.8 | -0.2 ± 6.4 | - | 2.2 ± 6.6 | -0.5 ± 6.1 | 0.9 ± 6.9 |
| LM with baseline wasting | 45.0 ± 4.8 | 47.8 ±5.5 | 48.0 ± 5.7 | 47.5 ± 5.6 | 36.4 ± 2.6 | 38.4 ±3.3 | 38.5 ±3.0 | 38.9 ±3.2 |
| ^1^Percent change in LM with baseline wasting | - | 6.6 ± 6.3 | -0.3 ± 4.8 | 0.4 ± 4.1 | - | 6.3 ± 4.6 | -1.5 ± 4.6 | 1.2 ± 4.3 |
| **Fat mass (FM)** |  |  |  |  |  |  |  |  |
| FM with no baseline wasting | 7.1 ± 1.8 | 8.0 ± 2.6 | 9.2 ± 3.1 | 8.8 ± 3.2 | 16.5 ± 6.5 | 15.9 ± 6.5 | 18.9 ±7.3 | 20.1 ± 7.5 |
| ^1^Percent change in FM with no baseline wasting | - | 15.4 ± 33.3 | 23.0 ± 41.9 | 3.5 ± 33.1 | - | -1.2 ± 23.8 | 19.7 ± 27.7 | 6.5 ± 23.6 |
| FM with baseline wasting | 3.3 ± 1.3 | 5.6 ± 2.6 | 6.4 ± 2.9 | 7.3 ± 3.8 | 5.9 ± 2.4 | 7.5 ± 3.3 | 10.5 ± 4.0 | 11.7 ± 3.2 |
| ^1^Percent change in FM with baseline wasting | - | 62.9 ± 66.8 | 14.6 ± 44.4 | 6.6 ± 34.7 | - | 28.3 ± 49.0 | 33.9 ± 49.7 | 22.8 ± 50.0 |
| **Body mass index (BMI)** |  |  |  |  |  |  |  |  |
| BMI with no baseline wasting | 20.2 ± 1.5 | 21.3 ± 1.5 | 21.9 ± 2.0 | 22.1 ± 2.3 | 21.7 ± 2.9 | 22.4 ± 2.8 | 23.4 ± 3.2 | 23.8 ± 3.6 |
| ^1^Percent change in BMI with no baseline wasting | - | 5.6 ± 5.3 | 2.5 ± 6.3 | 0.9 ± 4.5 | - | 4.1 ± 6.5 | 3.4 ± 8.1 | 1.5 ± 7.1 |
| BMI with baseline wasting | 17.0 ± 1.3 | 18.4 ± 1.6 | 18.7 ± 1.7 | 19.1 ± 2.0 | 16.7 ± 1.2 | 18.2 ± 1.8 | 19.6 ± 2.0 | 19.8 ± 2.3 |
| ^1^Percent change in BMI with baseline wasting | - | 8.4 ± 12.4 | 2.2 ± 7.0 | 2.3 ± 7.9 | - | 8.9 ± 8.7 | 6.2 ± 10.1 | 1.5 ± 10.6 |

^1^Percent change obtained by difference between observation for month 0 from month 3, month 3 from month 12, and month 12 from month 24, expressed as percentage .
